# Fixation of CO_2_ along with bromopyridines on a silver electrode

**DOI:** 10.1098/rsos.180897

**Published:** 2018-08-15

**Authors:** Yingtian Zhang, Shuxian Yu, Peipei Luo, Shisong Xu, Xianxi Zhang, Huawei Zhou, Jiyuan Du, Jie Yang, Nana Xin, Yuxia Kong, Junhai Liu, Baoli Chen, Jiaxing Lu

**Affiliations:** 1 Shandong Provincial Key Laboratory of Chemical Energy Storage and Novel Cell Technology, School of Chemistry and Chemical Engineering, Liaocheng University, Liaocheng 252059, People's Republic of China; 2 Shanghai Key Laboratory of Green Chemistry and Chemical Processes, School of Chemistry and Molecular Engineering, East China Normal University, Shanghai 200062, People's Republic of China

**Keywords:** CO_2_, electrocarboxylation, bromopyridines, silver electrode

## Abstract

Resulting from the drastic increase of atmospheric CO_2_ concentration day by day, global warming has become a serious environmental issue nowadays. The fixation of CO_2_ to obtain desirable, economically competitive chemicals has recently received considerable attention. This work investigates the fixation of CO_2_ along with three bromopyridines via a facile electrochemical method using a silver cathode to synthesize picolinic acids, which are important industrial and fine chemicals. Cyclic voltammetry is employed to investigate the cyclic voltammetric behaviour of bromopyridines. In addition, systematic study is conducted to study the relationships between the picolinic acids' yield and the electrolysis conditions and intrinsic parameters. The results show that the target picolinic acids' yields are strongly dependent on various conditions such as solvent, supporting electrolyte, current density, cathode material, charge passed, temperature and the nature of the substrates. Moreover, in the studied electrode materials such as Ag, Ni, Ti, Pt and GC, electrolysis and cyclic voltammetry show that Ag has a good electrocatalytic effect on the reduction and carboxylation of bromopyridine. This facile electrochemical route for fixation of CO_2_ provides an indispensable reference for the conversion and utilization of CO_2_ under mild conditions.

## 1. Introduction

Nowadays, the sustainable development of society and its related ecological environment, resources and economy have become the focus of the international community. But, global warming has been becoming a serious environmental problem due to the increasing concentration of atmospheric CO_2_, and hence how to effectively convert CO_2_ has become an urgent problem for chemists [1–5].

In contrast to toxic carbon monoxide and phosgene, CO_2_ is a renewable and environmentally friendly C1-organic building block [6–9]. However, owing to the intrinsic high thermodynamic stability of CO_2_, the facile reduction of CO_2_ has always been a challenge. Fortunately, electrochemical technology can reduce CO_2_ at normal temperature and atmospheric pressure [10–13]. So far, a lot of attention has been drawn to the study of the fixation of CO_2_ along with various substrates including epoxides [14], alcohols [15], imines [16–19], ketones [16–19], alkenes [20,21], dienes [22–24], alkynes [25] and halides [26–29]. In addition, electroreduction of organic halides is an extremely facile method to generate active anions which can readily activate CO_2_ to ensure that a carboxylic functional group is introduced into the organic centre while leaving intact the original carbon skeleton. That is why much attention is drawn to the study of the electrocarboxylation of organic halides [30–33]. Furthermore, some of these reaction pathways are particularly important for producing fine chemicals such as anti-inflammatory drugs. Catalytic systems based on transition-metal complexes, such as nickel [34], palladium [35] and cobalt complexes [36], have been proposed to increase the yield of the corresponding carboxylic acid. Electrode material with excellent catalytic properties can effectively improve the reaction, so mercury was once the preferred electrode material. However, it is now abandoned for being unfriendly to the environment. At present, the silver (Ag) electrode has been recognized as a useful catalyst for the electroreduction of organic halides [27,28,37–39]. Moreover, although electrocarboxylation with regard to organic halides has gained popularity in recent times, only a small number of reports involve the electrochemical reduction of heterocyclic halides along with CO_2_ [40]. Electrochemical carboxylation of heterocyclic halides is one of the most useful methods for obtaining heterocyclic carboxylic acids, which are a class of important compounds, and some of them are of meaning to the industry of fine chemicals such as anti-inflammatory drugs [41–43]. From the perspective of applications, high yields will greatly reduce production costs. To make the electrosynthesis of heterocyclic carboxylic acid industrially and commercially feasible, it is necessary to develop a low-cost, environmentally friendly, high-yield synthetic route.

**Scheme 1.**
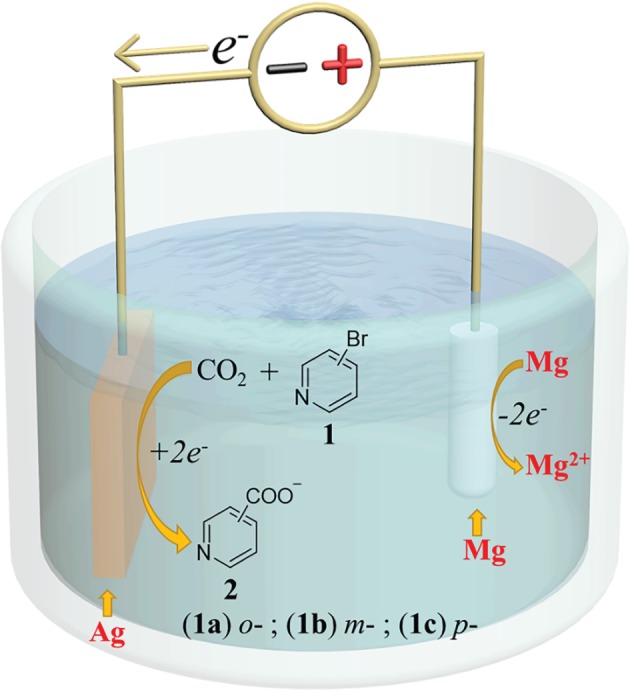
The Schematic diagram of electrocarboxylation of bromopyridines.

In this paper, we study the electrosynthesis of picolinic acids (**2**) from three bromopyridines (**1a, 1b, 1c**) and CO_2_ on Ag electrodes (scheme 1). To optimize the yield of **2**, on the one hand, the influence of a series of synthesis conditions on the electrocarboxylation of **1a** is investigated; on the other hand, the influence of the position of C–Br bond on the pyridine ring on the electrocarboxylation of **1,** which has not been reported previously, is the other major focus of our research. This study is to establish a facile electrochemical method for introducing CO_2_ into **1** using Ag as a cathode to yield **2** under mild conditions, providing a more effective and environmentally friendly method for the fixation of CO_2_.

## 2. Experimental

### 2.1. Chemicals

*Ortho*-bromopyridine (**1a**) and *meta*-bromopyridine (**1b**) are commercially obtained from J&K. *para*-bromopyridine (**1c**) is commercially available from Shanghai Lanke Medical Technology. Acetonitrile (MeCN) and *N*,*N*-dimethylformamide (DMF), which are kept over 4Å molecular sieves, are commercially obtained from Sinopharm Chemical. All other reagents are used without extra processing.

### 2.2. Product measurement

Cyclic voltammograms are recorded using a CHI 760E. Galvanostatic electrolysis is carried out using a DC-regulated power supply HY5003M equipped with a one-compartment electrochemical cell. The electrocarboxylation product yields based on the starting substrate are determined by high-performance liquid chromatography (HPLC) (Waters 505 pump) connected to a UV detector (Waters 2489) and a CenturySIL C18-EPS column.

### 2.3. Typical electroanalytical procedure

A typical electroanalytical experiment is carried out in 10 ml DMF with 0.1 M tetraethylammonium tetrafluoroborate (TEABF_4_) at 25°C in a one-compartment three-electrode electrochemical cell, along with glassy carbon (GC, diameter = 2 mm) or Ag (diameter = 2 mm) as a working electrode, Ag/AgI/0.1 M TBAI in DMF as a reference electrode and platinum spiral as an auxiliary electrode. All experiments are carried out at atmospheric pressure.

### 2.4. Typical electrosynthesis procedure

A typical galvanostatic electrolysis is performed in 10 ml of MeCN or DMF solution with 0.1 M supporting electrolyte and 0.1 M **1** in a one-compartment electrochemical cell, along with a sacrificial magnesium rod (Mg) anode and one of these cathodes: Ag (8 cm^2^) or Pt (4 cm^2^), Ni (8 cm^2^), GC (4 cm^2^), Ti (8 cm^2^). The system is always saturated with CO_2_ during the electrolysis process. After a certain amount of charge is passed through the cell, the current is switched off. At the end of the electrolysis, the solvent is distilled off *in vacuo*. The residue is hydrolysed in a mixture (24/76, v/v) of MeCN and a NaH_2_PO_4_/Na_2_HPO_4_ buffer at pH 6. Then the yields of **2** are determined by HPLC after appropriate dilution with the detection wavelength of 265 nm, and the eluent is a mixture (12/88, v/v) of MeCN and a NaH_2_PO_4_/Na_2_HPO_4_ buffer at pH 6.

## 3. Results and discussion

### 3.1. Electroanalytical measurements of *ortho*-bromopyridine

**Figure 1.**
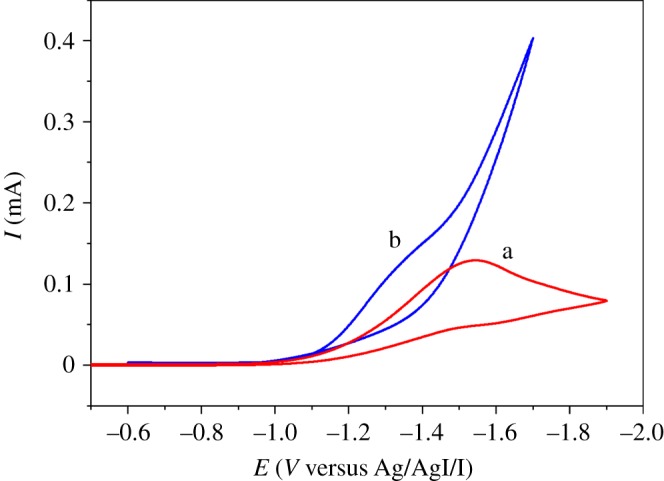
Cyclic voltammograms of 10 mM *o*-bromopyridine obtained in DMF + 0.1 M TEABF_4_ on a GC electrode in the (a) absence and (b) presence of CO_2_ with the scan rate of 0.1 V s^−1^.

Cyclic voltammograms recorded for reduction of *ortho*-bromopyridine (**1a**) on a GC electrode in DMF with 0.1 M TEABF_4_ are depicted in figure 1. As shown in curve a of Figure 1, electroreduction of **1a** causes a single irreversible cathodic peak at −1.55 V in the region of −0.5 to −2.0 V under a N_2_ atmosphere with a scan rate of 0.1 V s^−1^, which corresponds to a two-electron reduction of the C–Br bond. The synthesis of pyridine, which is detected by gas chromatography–mass spectrometry (GC–MS), via potentiostatic electrolysis at −1.55 V under a N_2_ atmosphere, also confirms the result. In addition, the reduction peak currents are proportional to *v*^1/2^ (electronic supplementary material, figure S1), indicating that the electroreduction process is diffusion-controlled. When the CO_2_ is piped into the system (0.2 M) [44], different behaviour is observed (figure 1, curve b). The reduction peak potential moves more positively and the current increases, indicating that there is a rapid chemical reaction between the electroreduced intermediate and CO_2_.

### 3.2. Preparative scale electrolysis

**Scheme 2.**
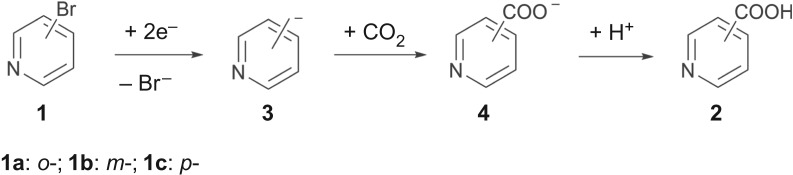
Electrocarboxylation of bromopyridines.

**1a** as a model molecule is first chosen to study the electrocarboxylation of **1** with a one-compartment electrochemical cell (scheme 2). A series of electrolytic reactions have been carried out to study the effect of different electrosynthetic conditions, such as solvent, supporting electrolyte, current density, cathode material, charge passed and temperature on the yield of carboxylation product **2**.

#### 3.2.1. Effect of solvent, supporting electrolyte and current density

**Scheme 3.**
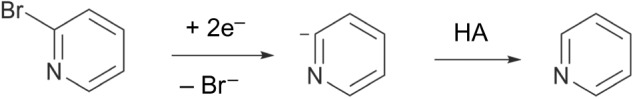
Electroreduction of *o*-bromopyridine with a hydrogen source.

Electrosynthesis is often influenced by the solvents [45]. In this paper, we investigate the electrocarboxylation of **1a** in a solvent of MeCN or DMF in order to study the effect of the solvents. As shown in table 1 (entries 1–4), **2a**'s yield in DMF is much higher than that in MeCN, despite the higher solubility of CO_2_ in MeCN [44]. Generally speaking, the electroreduction of aromatic bromides in protic media is likely to go through a reductive hydrogenation pathway [46]. The result that pyridine is detected by GC–MS via potentiostatic electrolysis at −1.55 V under a N_2_ atmosphere (in §3.1) indicates that the intermediate that resulted from two-electron reduction of **1a** would go through a hydrogenation step described in scheme 3 in the process of the reduction of **1a** just as the electroreduction of aromatic bromides in protic solvent, and yet scheme 3 is the competitive reaction of the carboxylation reaction described in scheme 2. When compared with DMF, MeCN is a more powerful proton donor [18,41], so it is more beneficial to the formation of the corresponding hydrogenated product than DMF during the reaction according to scheme 3. Besides, to further investigate the effect of a proton donor on the electrocarboxylation reaction, a certain amount of water is deliberately added into the system (entry 5). As shown in table 1 (entry 2 and entry 5), **2a**'s yield decreases from 55.0% to 20.6% when 0.1 M water is added into the system, which indicates that a proton donor has a greater impact on the reaction. In addition, DMF is a better solvent than acetonitrile, and it facilitates the dissolution of the products. In the electrolysis process, we observe that the DMF electrolyte is generally a transparent and clear liquid, while the MeCN electrolyte is turbid. In general, turbid electrolytes can affect the mass transfer process of the system, and then affect the electrocarboxylation reaction here. Moreover, we also observe that some undissolved substances are attached to the electrode surface in the electrolysis process and the electrolysis voltage is higher when MeCN is used as a solvent in the experiment, and these two phenomena are either detrimental to the reaction or are detrimental to energy use. Therefore, combining the electrolysis results and the solubility limit of reagent or product, the most suitable solvent in this study is DMF.

**Table 1.** Effect of solvent, supporting electrolyte and current density on the fixation of CO_2_ along with *o*-bromopyridine.^a^

entry	solvent	supporting electrolyte	current density (mA cm^−2^)	yield^c^ of **2a** (%)
1	MeCN	TBABr	8	16.4
2	DMF	TBABr	8	55.0
3	MeCN	TEABr	8	13.6
4	DMF	TEABr	8	46.2
5	DMF^b^	TBABr	8	20.6
6	DMF	TBACl	8	49.6
7	DMF	TBAI	8	50.8
8	DMF	TEACl	8	36.6
9	DMF	TEAI	8	38.6
10	DMF	TEABF_4_	8	41.4
11	DMF	TBABr	4	49.0
12	DMF	TBABr	6	50.6
13	DMF	TBABr	9	59.0
14	DMF	TBABr	10	55.6
15	DMF	TBABr	12	48.2
16	DMF	TBABr	16	42.6

^a^Electrolytic conditions: 10 ml of solvent, 0.1 M supporting electrolyte concentration, 0.1 M *o*-bromopyridine, Ag cathode, Mg anode, 0°C, 2 F mol^−1^ charge passed, 1 atm CO_2_.

^b^0.1 M water is added to the system.

^c^The yield calculated with respect to the starting substrate is determined by HPLC.

Next, we study the effect of the supporting electrolyte on the reaction. The results are summarized in table 1 (entries 2, 4, 6–10). The best supporting electrolyte is tetrabutylammonium bromide (TBABr) with 55.0% electrocarboxylation yield. It is notable that with the same anion of Cl^−^, Br^−^, I^−^, respectively, the electrolysis concerning the TBA^+^ cation gives higher carboxylation yield than that involved with TEA^+^ (table 1, entries 2, 4, 6–9), showing that the cations may impact the synthesis. It is known that protonation of *o*-bromopyridine described in scheme 3 is the competitive reaction of the carboxylation of *o*-bromopyridine described in scheme 2. In fact, the tetraalkylammonium cation (TRA^+^) can act as a proton donor strongly depending on the length of the alkyl chain via Hoffman elimination [47,48], and the TEA^+^ cation is a much better proton donor than the TBA^+^ cation [49]. So, when tetraethylammonium (TEA^+^) salt acts as a supporting electrolyte, it is more beneficial to the formation of the corresponding hydrogenated product than tetrabutylammonium (TBA^+^) salt during the reaction. For that reason, the electrocarboxylation process reported here gives higher picolinic acid yields in the presence of the TBA^+^ cation than the TEA^+^ cation (table 1, entries 2, 4, 6–9). In addition, in the same TRA^+^ cases, the carboxylation yield decreases in the following sequence: Br^−^ > I^−^ > Cl^−^ (table 1, entries 2, 4, 6–9).

The current density affects the reaction. Higher or lower current densities lead to lower electrocarboxylation yield owing to the significant contribution from the undesired Faradaic process. As shown from the results summarized in entry 2 and entries 11–16 of table 1, with the increase of current density from 4 to 9 mA cm^−2^, the yield is increased from 49.0% to 59.0%; but the electrocarboxylation yield declines when the current density is raised over 9 mA cm^−2^.

#### 3.2.2. Effect of cathode material, charge passed and temperature

**Figure 2.**
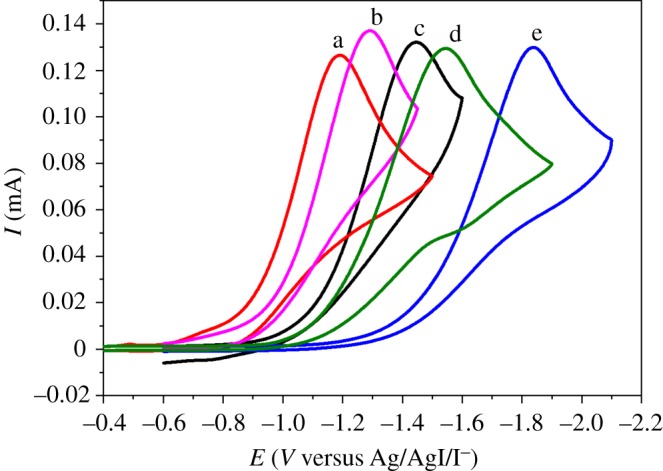
Cyclic voltammograms of 10 mM *o*-bromopyridine obtained in DMF + 0.1 M TEABF_4_ with the scan rate of 0.1 V s^−1^ on different electrodes: (a) Ag; (b) Pt; (c) Ni; (d) GC; (e) Ti.

The nature of cathode material also strongly affects the reaction. As shown from the results in table 2 (entries 1–5), the best cathode material is Ag with 59.0% electrocarboxylation yield (table 2, entry 1). Table 2 (entries 1–5) also shows that the yields decrease according to the cathode materials in the following sequence: Ag (59.0%) > Pt (36.2%) > Ni (30.4%) > GC (18.6%) > Ti (10.2%). The yields are related to the reduction potentials of **1a** on the electrodes used: Ag (−1.19 V, curve a of figure 2) > Pt (−1.29 V, curve b of figure 2) > Ni (−1.45 V, curve c of figure 2) > GC (−1.55 V, curve d of figure 2) > Ti (−1.84 V, curve e of figure 2). Both electrolysis and cyclic voltammetry indicate that Ag displays outstanding electrocatalytic activities with regard to the reduction and carboxylation of **1a**.

**Table 2.** Effect of cathode material, charge passed and temperature on the fixation of CO_2_ along with *o*-bromopyridine.^a^

entry	cathode material	charge passed (F mol^−1^)	temperature (°C)	yield^b^ of **2a** (%)
1	Ag	2.0	0	59.0
2	Ni	2.0	0	30.4
3	Ti	2.0	0	10.2
4	Pt	2.0	0	36.2
5	GC	2.0	0	18.6
6	Ag	1.0	0	15.6
7	Ag	1.5	0	31.4
8	Ag	2.5	0	44.2
9	Ag	3.5	0	35.8
10	Ag	2.0	−5	35.8
11	Ag	2.0	5	32.0
12	Ag	2.0	25	21.6

^a^Electrolytic conditions: 10 ml DMF, 0.1 M TBABr, 0.1 M *o*-bromopyridine, Mg anode, 9 mA cm^−2^ current density, 1 atm CO_2_.

^b^The yield calculated with respect to the starting substrate is determined by HPLC.

Then the effect of the charge passed is discussed. The results are presented in table 2 (entries 1, 6–9). When the charge passed is increased from 1.0 to 2.0 F mol^−1^, the electrocarboxylation yield increases linearly; however, the yield decreases when the charge passed is raised over 2.0 F mol^−1^. Therefore, the best choice is 2.0 F mol^−1^ of **1a**.

The influence of the temperature is also complicated. In general, the temperature affects the solubility of CO_2_ in the solvent, as well as the nature of the thermodynamics and kinetics of carboxylation. On the one hand, decreasing the temperature will increase the solubility of CO_2_ in the solvent [44]; on the other hand, decreasing the temperature may decrease the activity of the reactants. To study the influence of temperature, electrolysis is performed at diverse temperatures. The results are given in table 2 (entries 1, 10–12). When the temperature is raised from −5°C to 0°C, the yield increases (table 2, entries 1, 10), but the yield reduces when the temperature is further raised (table 2, entries 1, 11, 12). So, 0°C is the optimal temperature.

#### 3.2.3. Influence of the nature of substrates

To test the validity and universality of the provided electrochemical route for fixation of CO_2_ and investigate the influence of the position of the C–Br bond on the pyridine ring on the reaction, the investigation is expanded to *m*-bromopyridine (**1b**) and *p*-bromopyridine (**1c**) under the optimized electrolytic conditions (table 2, entry 1). As shown from the results in table 3, CO_2_ incorporation into **1** obtaining the corresponding carboxylation product **2** with good electrocarboxylation yields is successfully completed in all cases and the yield of **2** increases from 59.0% to 65.8% in the following sequence: *o*-picolinic acid < *m*-picolinic acid < *p*-picolinic acid.

**Table 3.** Fixation of CO_2_ along with bromopyridines.^a^

entry	substrate	yield^b^ of **2** (%)
1	*o*-bromopyridine (**1a**)	59.0
2	*m*-bromopyridine (**1b**)	62.6
3	*p*-bromopyridine (**1c**)	65.8

^a^Electrolytic conditions: 10 ml DMF, 0.1 M TBABr, 0.1 M bromopyridine, Ag cathode, Mg anode, 9 mA cm^−2^ current density, 0°C, 2 F mol^−1^ charge passed, 1 atm CO_2_.

^b^The yield calculated with respect to the starting substrate is determined by HPLC.

**Figure 3.**
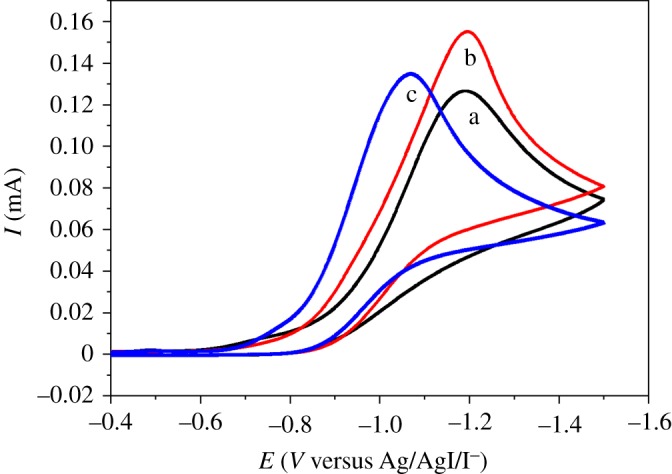
Cyclic voltammograms of 10 mM bromopyridine conducted on a Ag electrode in DMF + 0.1 M TEABF_4_ with the scan rate of 0.1 V s^−1^: (a) *o*-bromopyridine; (b) *m*-bromopyridine; (c) *p*-bromopyridine.

In addition, the cyclic voltammograms are also extended to *m*-bromopyridine and *p*-bromopyridine. As revealed in figure 3, an irreversible reduction peak for the two-electron transfer process of the C–Br bond is obtained for all the three bromopyridines on the Ag electrode. The reaction peak potential of *p*-bromopyridine is significantly more positive than that of *o*-bromopyridine and *m*-bromopyridine, showing that *p*-bromopyridine is more easily reduced than *o*-bromopyridine and *m*-bromopyridine. In addition, the peak potentials of *o*-bromopyridine and *m*-bromopyridine are very close, except that the peak current of *m*-bromopyridine is higher than that of *o*-bromopyridine, and this may be due to the fact that the diffusion coefficient of *m*-bromopyridine in the system is larger than that of *o*-bromopyridine. In combination with the previous electrolysis results (table 3) we can know that the electrochemical route for the fixation of CO_2_ works well for all the **1,** and *p*-bromopyridine with the most positive reduction potential achieves optimal performance in CO_2_ fixation.

## 4. Conclusion

In conclusion, the important pharmaceutical intermediate picolinic acids (**2**) are synthesized by electrocarboxylation from three bromopyridines (**1**) and CO_2_ using a Ag electrode. The electrolysis experiment is conducted under mild conditions with a one-compartment electrochemical cell. The effect of various electrolysis conditions and intrinsic properties on the fixation of CO_2_ along with **1** has been investigated to improve the yield. After optimizing the synthetic parameters, the target **2** with good yields (59.0–65.8%) is achieved in the DMF-TBABr electrolyte with a current density of 9 mA cm^−2^ and an electric charge of 2 F mol^−1^ on a Ag electrode at 0°C. In addition, among the five materials of Ag, Ni, Ti, Pt and GC, both electrolysis and cyclic voltammograms show that Ag has the best electrocatalytic performance for the reduction and carboxylation of **1**. Moreover, the position of the C–Br bond on the pyridine ring would affect the electrocarboxylation reaction. Among the **1** investigated, both cyclic voltammetry and preparative electrolysis indicate *para*-bromopyridine achieves optimal performance in CO_2_ fixation. This research is of significance for fundamental research and practical applications of CO_2_ fixation and synthesis of pharmaceutical intermediates by a simple and efficient means.

## Supplementary Material

Cyclic voltammograms of 10 mM o–bromopyridine on a GC electrode in DMF with 0.1 M TEABF4 at (a) v=0.1 V/s, (b) 0.2 V/s, (c) 0.3 V/s, (d) 0.4 V/s, (e) 0.5 V/s, (f) 0.6 V/s, (g) 0.7 V/s, (h) 0.8 V/s and (i) 0.9 V/s.

## Data accessibility

All data used in this article are present in the article and its electronic supplementary material.

## References

[1] Gao S, Lin Y, Jiao X, Sun Y, Luo Q, Zhang W, Li D, Yang J, Xie Y 2016 Partially oxidized atomic cobalt layers for carbon dioxide electroreduction to liquid fuel. Nature 529, 68–71. (10.1038/nature16455) 26738592

[2] Poland SJ, Darensbourg DJ 2017 A quest for polycarbonates provided via sustainable epoxide/CO_2_ copolymerization processes. Green Chem. 19, 4990–5011. (10.1039/C7GC02560B)

[3] Diercks CS, Lin S, Kornienko N, Kapustin EA, Nichols EM, Zhu C, Zhao Y, Chang CJ, Yaghi OM 2018 Reticular electronic tuning of porphyrin active sites in covalent organic frameworks for electrocatalytic carbon dioxide reduction. J. Am. Chem. Soc. 140, 1116–1122. (10.1021/jacs.7b11940) 29284263

[4] Clark EL, Hahn C, Jaramillo TF, Bell AT 2017 Electrochemical CO_2_ reduction over compressively strained CuAg surface alloys with enhanced multi-carbon oxygenate selectivity. J. Am. Chem. Soc. 139, 15 848–15 857. (10.1021/jacs.7b08607) 28988474

[5] Chang X, Wang T, Zhang P, Wei Y, Zhao J, Gong J 2016 Stable aqueous photoelectrochemical CO_2_ reduction by a Cu_2_O dark cathode with improved selectivity for carbonaceous products. Angew. Chem. Int. Ed. 55, 8840–8845. (10.1002/anie.201602973) 27199242

[6] Jiang K et al. 2018 Isolated Ni single atoms in graphene nanosheets for high-performance CO_2_ reduction. Energy Environ. Sci. 11, 893–903. (10.1039/C7EE03245E)

[7] Olivo A, Ghedini E, Pascalicchio P, Manzoli M, Cruciani G, Signoretto M 2018 Sustainable carbon dioxide photoreduction by a cooperative effect of reactor design and titania metal promotion. Catalysts 8, 41 (10.3390/catal8010041)

[8] Liu Q, Wu L, Jackstell R, Beller M 2015 Using carbon dioxide as a building block in organic synthesis. Nat. Commun. 6, 12394 (10.1038/ncomms6933) 25600683

[9] Li X et al. 2017 Exclusive Ni–N_4_ sites realize near-unity CO selectivity for electrochemical CO_2_ reduction. J. Am. Chem. Soc. 139, 14 889–14 892. (10.1021/jacs.7b09074) 28992701

[10] Sung S, Kumar D, Gil-Sepulcre M, Nippe M 2017 Electrocatalytic CO_2_ reduction by imidazolium-functionalized molecular catalysts. J. Am. Chem. Soc. 139, 13 993–13 996. (10.1021/jacs.7b07709) 28921978

[11] Jiao Y, Zheng Y, Chen P, Jaroniec M, Qiao SZ 2017 Molecular scaffolding strategy with synergistic active centers to facilitate electrocatalytic CO_2_ reduction to hydrocarbon/alcohol. J. Am. Chem. Soc. 139, 18 093–18 100. (10.1021/jacs.7b10817) 29151346

[12] Qiao J, Jiang P, Liu J, Zhang J 2014 Formation of Cu nanostructured electrode surfaces by an annealing–electroreduction procedure to achieve high-efficiency CO_2_ electroreduction. Electrochem. Commun. 38, 8–11. (10.1016/j.elecom.2013.10.023)

[13] Yuan J, Liu L, Guo RR, Zeng S, Wang H, Lu JX 2017 Electroreduction of CO_2_ into ethanol over an active catalyst: copper supported on titania. Catalysts 7, 220 (10.3390/catal7070220)

[14] Xiao Y, Chen BL, Yang HP, Wang H, Lu JX 2014 Electrosynthesis of enantiomerically pure cyclic carbonates from CO_2_ and chiral epoxides. Electrochem. Commun. 43, 71–74. (10.1016/j.elecom.2014.03.012)

[15] Wu LX, Wang H, Xiao Y, Tu ZY, Ding BB, Lu JX 2012 Synthesis of dialkyl carbonates from CO_2_ and alcohols via electrogenerated N-heterocyclic carbenes. Electrochem. Commun. 25, 116–118. (10.1016/j.elecom.2012.09.028)

[16] Feroci M, Orsini M, Rossi L, Sotgiu G, Inesi A 2007 Electrochemically promoted C–N bond formation from amines and CO_2_ in ionic liquid BMIm-BF_4_: synthesis of carbamates. J. Org. Chem. 72, 200–203. (10.1021/jo061997c) 17194100

[17] Feng Q, Huang K, Liu S, Yu J, Liu F 2011 Electrocatalytic carboxylation of aromatic ketones with carbon dioxide in ionic liquid 1-butyl-3-methylimidazoliumtetrafluoborate to α-hydroxy-carboxylic acid methyl ester. Electrochim. Acta 56, 5137–5141. (10.1016/j.electacta.2011.03.061)

[18] Zhao SF, Wang H, Lan YC, Liu X, Lu JX, Zhang J 2012 Influences of the operative parameters and the nature of the substrate on the electrocarboxylation of benzophenones. J. Electroanal. Chem. 664, 105–110. (10.1016/j.jelechem.2011.11.001) PMC3284772 22368535

[19] Chen B-L, Tu Z-Y, Zhu H-W, Sun W-W, Wang H, Lu J-X 2014 CO_2_ as a C1-organic building block: enantioselective electrocarboxylation of aromatic ketones with CO_2_ catalyzed by cinchona alkaloids under mild conditions. Electrochim. Acta 116, 475–483. (10.1016/j.electacta.2013.11.001)

[20] Wang H, Zhang G, Liu Y, Luo Y, Lu J 2007 Electrocarboxylation of activated olefins in ionic liquid BMIMBF4. Electrochem. Commun. 9, 2235–2239. (10.1016/j.elecom.2007.06.031)

[21] Wang H, Zhang K, Liu YZ, Lin MY, Lu JX 2008 Electrochemical carboxylation of cinnamate esters in MeCN. Tetrahedron 64, 314–318. (10.1016/j.tet.2007.10.104)

[22] Li CH, Yuan GQ, Ji XC, Wang XJ, Ye JS, Jiang HF 2011 Highly regioselective electrochemical synthesis of dioic acids from dienes and carbon dioxide. Electrochim. Acta 56, 1529–1534. (10.1016/j.electacta.2010.06.057)

[23] Zhang K, Xiao YJ, Lan YC, Zhu MX, Wang HA, Lu JX 2010 Electrochemical reduction of aliphatic conjugated dienes in the presence of carbon dioxide. Electrochem. Commun. 12, 1698–1702. (10.1016/j.elecom.2010.09.028)

[24] Steinmann SN, Michel C, Schwiedernoch R, Wu MJ, Sautet P 2016 Electro-carboxylation of butadiene and ethene over Pt and Ni catalysts. J. Catal. 343, 240–247. (10.1016/j.jcat.2016.01.008)

[25] Köster F, Dinjus E, Duñach E 2001 Electrochemical selective incorporation of CO_2_ into terminal alkynes and diynes. Eur. J. Org. Chem. 2001, 2507–2511. (10.1002/1099-0690(200107)2001:13%3C2507::AID-EJOC2507%3E3.0.CO;2-P)

[26] Chen BL, Zhu HW, Xiao Y, Sun QL, Wang H, Lu JX 2014 Asymmetric electrocarboxylation of 1-phenylethyl chloride catalyzed by electrogenerated chiral [CoI(salen)]-complex. Electrochem. Commun. 42, 55–59. (10.1016/j.elecom.2014.02.009)

[27] Luo PP et al. 2017 Electrocarboxylation of dichlorobenzenes on a silver electrode in DMF. Catalysts 7, 274 (10.3390/catal7090274)

[28] Niu DF, Xiao LP, Zhang AJ, Zhang GR, Tan QY, Lu JX 2008 Electrocatalytic carboxylation of aliphatic halides at silver cathode in acetonitrile. Tetrahedron 64, 10 517–10 520. (10.1016/j.tet.2008.08.093)

[29] Wang HM, Sui GJ, Wu D, Feng Q, Wang H, Lu JX 2016 Selective electrocarboxylation of bromostyrene at silver cathode in DMF. Tetrahedron 72, 968–972. (10.1016/j.tet.2015.12.066)

[30] Scialdone O, Galia A, Belfiore C, Filardo G, Silvestri G 2004 Influence of the experimental system and optimization of the selectivity for the electrocarboxylation of chloroacetonitrile to cyanoacetic acid. Ind. Eng. Chem. Res. 43, 5006–5014. (10.1021/ie034275%2B)

[31] Scialdone O, Galia A, Silvestri G, Amatore C, Thouin L, Verpeaux JN 2006 Electrocarboxylation of benzyl halides through redox catalysis on the preparative scale. Chemistry 12, 7433–7447. (10.1002/chem.200501499) 16850513

[32] Sui G-J, Sun Q-L, Wu D, Meng W-J, Wang H, Lu J-X 2016 Electrocatalytic reduction of PhCH_2_ Cl on Ag-ZSM-5 zeolite modified electrode. RSC Adv. 6, 63 493–63 496. (10.1039/C6RA09141E)

[33] Wang H, He L, Sui G-J, Lu J-X 2015 Electrocatalytic reduction of PhCH_2_ Br on a Ag-Y zeolite modified electrode. RSC Adv. 5, 42 663–42 665. (10.1039/C5RA03970C)

[34] Gennaro A, Isse AA, Maran F 2001 Nickel(I)(salen)-electrocatalyzed reduction of benzyl chlorides in the presence of carbon dioxide. J. Electroanal. Chem. 507, 124–134. (10.1016/S0022-0728(01)00373-4)

[35] Damodar J, Krishna Mohan SR, Jayarama Reddy SR 2001 Synthesis of 2-arylpropionic acids by electrocarboxylation of benzylchlorides catalysed by PdCl2(PPh3)2. Electrochem. Commun. 3, 762–766. (10.1016/S1388-2481(01)00263-6)

[36] Fabre PL, Reynes O 2010 Electrocarboxylation of chloroacetonitrile mediated by electrogenerated cobalt(I) phenanthroline. Electrochem. Commun. 12, 1360–1362. (10.1016/j.elecom.2010.07.020)

[37] Isse AA, Gennaro A 2002 Electrocatalytic carboxylation of benzyl chlorides at silver cathodes in acetonitrile. Chem. Commun. 2798–2799. (10.1039/b206746c) 12478752

[38] Scialdone O, Guarisco C, Galia A, Herbois R 2010 Electroreduction of aliphatic chlorides at silver cathodes in water. J. Electroanal. Chem. 641, 14–22. (10.1016/j.jelechem.2010.01.018)

[39] Scialdone O, Galia A, Filardo G, Isse AA, Gennaro A 2008 Electrocatalytic carboxylation of chloroacetonitrile at a silver cathode for the synthesis of cyanoacetic acid. Electrochim. Acta 54, 634–642. (10.1016/j.electacta.2008.07.012)

[40] Ramesh Raju R, Krishna Mohan S, Jayarama Reddy S 2003 Electroorganic synthesis of 6-aminonicotinic acid from 2-amino-5-chloropyridine. Tetrahedron Lett. 44, 4133–4135. (10.1016/S0040-4039(03)00816-5)

[41] Gennaro A, Sánchez-Sánchez CM, Isse AA, Montiel V 2004 Electrocatalytic synthesis of 6-aminonicotinic acid at silver cathodes under mild conditions. Electrochem. Commun. 6, 627–631. (10.1016/j.elecom.2004.04.019)

[42] Feng QJ, Huang KL, Liu SQ, Wang XY 2010 Electrocatalytic carboxylation of 2-amino-5-bromopyridine with CO_2_ in ionic liquid 1-butyl-3-methyllimidazoliumtetrafluoborate to 6-aminonicotinic acid. Electrochim. Acta 55, 5741–5745. (10.1016/j.electacta.2010.05.010)

[43] Ghobadi K, Zare HR, Khoshro H, Gorji A 2016 Communication—electrosynthesis of isonicotinic acid via indirect electrochemical reduction of pyridine in the presence of CO_2_. J. Electrochem. Soc. 163, H240–H242. (10.1149/2.1121603jes)

[44] Gennaro A, Isse AA, Vianello E 1990 Solubility and electrochemical determination of CO_2_ in some dipolar aprotic solvents. J. Electroanal. Chem. 289, 203–215. (10.1016/0022-0728(90)87217-8)

[45] Izutsu K 2009 Electrochemistry in nonaqueous solutions, 2nd edn Hoboken, NJ: Wiley-VCH.

[46] Isse AA, Durante C, Gennaro A 2011 One-pot synthesis of benzoic acid by electrocatalytic reduction of bromobenzene in the presence of CO_2_. Electrochem. Commun. 13, 810–813. (10.1016/j.elecom.2011.05.009)

[47] Vieira KL, Mubarak MS, Peters DG 1984 Use of deuterium labeling to assess the roles of tetramethylammonium cation, dimethylformamide, and water as proton donors for electrogenerated tert-butyl carbanions: evidence for the formation of an ylide (trimethylammonium methylide). J. Am. Chem. Soc. 106, 5372–5373. (10.1021/ja00330a068)

[48] Vieira KL, Peters DG 1986 Electrolytic reduction of tert-butyl bromide at mercury cathodes in dimethylformamide. J. Org. Chem. 51, 1231–1239. (10.1021/jo00358a013)

[49] Dahm CE, Peters DG 1996 Electrochemical reduction of tetraalkylammonium tetrafluoroborates at carbon cathodes in dimethylformamide. J. Electroanal. Chem. 402, 91–96. (10.1016/0022-0728(95)04209-1)

