# Supplementary material for: Fixation of CO2 along with bromopyridines on a silver electrode
Source: R Soc Open Sci. 2018 Aug 15;5(8):180897. doi: 10.1098/rsos.180897 (PMC6124031; doi:10.1098/rsos.180897)
Supplement: Cyclic voltammograms of 10 mM o–bromopyridine on a GC electrode in DMF with 0.1 M TEABF4 at (a) v=0.1 V/s, (b) 0.2 V/s, (c) 0.3 V/s, (d) 0.4 V/s, (e) 0.5 V/s, (f) 0.6 V/s, (g) 0.7 V/s, (h) 0.8 V/s and (i) 0.9 V/s. [file rsos180897supp1.docx]

Royal society open science

Electronic Supplementary Materials

Fixation of CO_2_ along with bromopyridines on a silver electrode

Yingtian Zhang^a^, Shuxian Yu^a^, Peipei Luo^a^, Shisong Xu^a^, Xianxi Zhang^a^, Huawei Zhou^a^, Jiyuan Du^a^, Jie Yang^a^, Nana Xin^a^, Yuxia Kong^a^, Junhai Liu^a^, Baoli Chen*_­_^a^ and Jiaxing Lu*^b^

^a^Shandong Provincial Key Laboratory of Chemical Energy Storage and Novel Cell Technology, College of Chemistry and Chemical Engineering, Liaocheng University, Liaocheng 252059, China

^b^Shanghai Key Laboratory of Green Chemistry and Chemical Processes, School of Chemistry and Molecular Engineering, East China Normal University, Shanghai200062, China

** Corresponding authors: E-mail: goodchenbaoli@163.com； E-mail:* [*jxlu@chem.ecnu.edu.cn*](mailto:jxlu@chem.ecnu.edu.cn)

**Figure S1** Cyclic voltammograms of 10 mM *o*–bromopyridine on a GC electrode in DMF with 0.1 M TEABF_4_ at (a) *v*=0.1 V/s, (b) 0.2 V/s, (c) 0.3 V/s, (d) 0.4 V/s, (e) 0.5 V/s, (f) 0.6 V/s, (g) 0.7 V/s, (h) 0.8 V/s and (i) 0.9 V/s.

**Table S1**

Effect of THF, MeOH and DMSO on the fixation of CO_2_ along with *o*–bromopyridine^a^

| Entry | Solvent | Yield^b^ of **2a** (%) |
| --- | --- | --- |
| 1 | THF | --- |
| 2 | MeOH | 1.9 |
| 3 | DMSO | --- |

^a^ Electrolytic conditions: 10 mL solvent, 0.1 M TBABr, 0.1 M *o*–bromopyridine, 8 mA/cm^2^, Ag cathode, Mg anode, 0 °C, 2 F/mol charge passed, 1 atm CO_2_

^b^ The yield based on starting substrate is determined by HPLC

**Table S2**

Fixation of CO_2_ along with *o*–bromopyridine and *o*–chloropyridine^a^

| Entry | Substrate | Yield^b^ of **2a** (%) |
| --- | --- | --- |
| 1 | *o*−bromopyridine | 55.0 |
| 2 | *o*–chloropyridine | 16.2 |

^a^ Electrolytic conditions: 10 mL DMF, 0.1 M TBABr, 0.1 M substrate, 8 mA/cm^2^, Ag cathode, Mg anode, 0 °C, 2 F/mol charge passed, 1 atm CO_2_

^b^ The yield based on starting substrate is determined by HPLC
